# A bioactive hydrogel integrating bFGF/VEGFA gene-loaded nanoparticles and platelet-rich plasma for accelerated full-thickness skin wound healing

**DOI:** 10.1371/journal.pone.0350087

**Published:** 2026-05-26

**Authors:** Yan Duan, Weixin Wang, Yuanyuan Jin, Ming Jiang, Bolin Wang, Jianle Chen, Yun Jiang

**Affiliations:** 1 Department of Burn and Plastic Surgery, Affiliated Hospital of Nantong University, Medical School of Nantong University, Nantong, Jiangsu, China; 2 Department of Thoracic Surgery, Affiliated Hospital of Nantong University, Medical School of Nantong University, Nantong, Jiangsu, China; 3 Department of Burn and Plastic Surgery, The First People’s Hospital of Kunshan, Suzhou, Jiangsu, China; Arizona State University, UNITED STATES OF AMERICA

## Abstract

**Research objectives:**

Full-thickness skin defects present substantial challenges to the healing process, arising from the loss of the dermal layer, inadequate vascular supply, and insufficient growth factor availability. Although previous studies have established that growth factors and platelet-rich plasma (PRP) individually promote tissue repair, the synergistic benefits of their combined application for achieving superior therapeutic outcomes remain unclear. Here, we introduce a novel composite biomaterial: a hydrogel integrating nanoparticles loaded with basic fibroblast growth factor (bFGF) and vascular endothelial growth factor A (VEGFA) genes (bFGF/VEGFA NPs) with PRP (termed bFGF/VEGFA@PRP hydrogel), engineered to enable the synergistic delivery of growth factors and bioactive components.

**Methods:**

In this study, we hypothesized that the bFGF/VEGFA@PRP hydrogel can promote wound healing. This hypothesis was tested through various experimental perspectives and methods including material characterization, wound healing rate, wound blood flow signal intensity, skin attachment, cell proliferation, apoptosis, collagen deposition, and growth factor levels.

**Results:**

In a rat cutaneous wound model, this hydrogel accelerated wound closure, enhanced local blood perfusion, and increased the density of skin appendages and collagen fibers. Mechanistic investigations revealed upregulated expression of angiogenic factors bFGF, VEGFA, and platelet and endothelial cell adhesion molecule 1 (CD31), the anti-apoptotic protein BCL2, the cell proliferation marker Ki67, and type III collagen (COL III) in the treated group.

**Conclusions:**

Collectively, these results demonstrate that the bFGF/VEGFA@PRP hydrogel promotes wound healing through the synergistic enhancement of growth factor expression, angiogenesis, and COL III deposition. The combination of gene therapy with PRP treatment was found to be more effective than either modality alone. This study establishes a promising therapeutic strategy for managing complex full-thickness skin injuries.

## 1. Introduction

Full-thickness skin defects, characterized by the complete loss of epidermis, dermis, and associated appendages (e.g., hair follicles, sweat glands), pose significant clinical challenges due to their lack of regeneration capacity driven by re-epithelialization alone [1,2]. Such injuries, commonly resulting from burns, trauma, or chronic diseases，frequently cause pathological scarring and functional impairment [3]. Therefore, strategies that promote healing outcomes should be developed to repair skin barrier and resist external invasion. Skin wound healing is a complex biological process. It involves dynamic regulation across multiple stages, including inflammation, cell proliferation, angiogenesis, and tissue remodeling [4]. Collagen deposition plays an important role in wound healing [5]. Thus, wound treatments should be designed to establish a balance of these factors. To date, several drugs have been developed and applied in the treatment of skin wounds.

Notably, wound healing is driven by interactions between various cell types (e.g., fibroblasts and endothelial cells) as well as growth factors, cytokines and enzymes [6,7]. Although growth factors such as basic fibroblast growth factor (bFGF) [8–10] and vascular endothelial growth factor A (VEGFA) [11,12] play crucial roles in angiogenesis and collagen deposition during wound repair [13], they undergo rapid enzymatic degradation in vivo and have short half-lives (typically 2–6 hours), which limits their clinical translation [14]. Similarly, Platelet-rich plasma (PRP) is an autologous reservoir of these mediators and has been widely used to accelerate re-epithelialization and neovascularization. However, Standard PRP preparations contain only physiological or sub-therapeutic levels of bFGF and VEGFA, significantly below the doses required to accelerate full-thickness skin closure in pre-clinical models [15]. Moreover, > 80% of these bioactive components are lost within the first 72 h after application, a burst-release profile that fails to cover the entire proliferative and remodeling phases of healing [16, 17]. Although both modalities individually enhance healing, they do not provide sustained production of growth factors, which are required for full-thickness defect regeneration. Gene therapy offers a promising alternative by enabling endogenous, sustained expression of therapeutic factors [18], yet an optimal delivery system integrating PRP’s bioactivity with controlled gene release remains unexplored.

To address these limitations, we propose a strategy: a bioactive hydrogel composite combining bFGF/VEGFA gene-loaded nanoparticles (that ensure sustained factor expression) and PRP (that provides immediate growth factor release). This design leverages (1) the protective capacity of polymeric nanoparticles to shield plasmids from nucleases and enable controlled gene delivery, which achieve sustained release of growth factors, and (2) to bridge the therapeutic gap during the initial lag phase between gene transfection and transgene expression, we incorporated PRP to cover the early stage of wound repair. Accumulating evidence has demonstrated that the bioactive constituents within PRP play pivotal roles in modulating the inflammatory phase of wound healing [19]. We hypothesize that this dual-phase system—immediate PRP-derived bioactivity followed by nanoparticle-mediated prolonged gene expression—is likely to cover the whole process of wound repair, thereby accelerating regeneration.

## 2. Methods and materials

### 2.1. Experimental animals and animal models

A total of 19 eight-week-old Sprague Dawley (SD) male rats, each weighing 250 g, were purchased from the Animal Experiment Center of Nantong University. These rats were allowed to adapt to the laboratory for a week in a 12-hour light/dark cycle environment controlled to a temperature of 25°C. They were also provided adequate water and food. To alleviate the pain of animals, we use anesthetics reasonably, and adopt the method of quickly dislocating the head and neck under anesthesia to euthanize rats and alleviate the pain of animals. All biological waste was disposed of according to institutional guidelines for hazardous waste. Ethics approval Animal experiments were approved by the Animal Experiment Ethics Committee of Nantong University (20230730−010).

The rats were anaesthetized with 2% sodium pentobarbital at a dose of 50 mg/kg, 30 minutes before the experiment. After the removal of hair of the back of rats, using depilatory creams, the area was disinfected with iodine povidone and the center of the rat’s back was marked with methylene blue. Then, use sterilized ophthalmic scissors to cut the skin along the mark, creating a 1 × 1 cm full-thickness skin defect, which reached the subcutaneous tissue and was about 1 mm deep.

### 2.2. Preparation of expression vectors

The vector plasmid Enhanced Green Fluorescent Protein N1 (pEGFP-N1) (C04001, Shanghai GeneChem Co., Ltd., Shanghai, China) carried enhanced green fluorescent protein (EGFP) to explore whether the complex was successfully transfected. Two overexpression plasmids named; pEGFP-VEGFA and pEGFP-BFGF, were prepared by inserting the open reading frames (ORFs) of VEGFA (GenBank Accession Number: NM_001171626) and bFGF (GenBank Accession Number: NM_002006) into the pEGFP-N 1 vector. Subsequently, gene sequencing was performed to confirm that the ORFs were successfully inserted into the vector.

### 2.3. Preparation of nanoparticles

Nanoparticles were produced by the probe ultrasound technology [20,21]. Initially, a polylactic acid hydroxy acetic acid copolymer (200 mg, 719897, Sigma-Aldrich, St. Louis, MO, USA) was dissolved in dichloromethane (DCM, 2 mL) at 25°C, and fully shaken to obtain a 10% polymer solution. 7% water-based polyvinyl alcohol (PVA, 363081, SigmaAldrich, St. Louis, MO, USA) solution was mixed with DCM solution and allowed to incubate for 3 minutes on an ice bath using a probe ultrasonic device. Subsequently, the emulsion was introduced into an aqueous 100 ml 1% PVA solution. The mixture was then sonicated for 4 minutes in an ice bath using the same probe ultrasonic instrument to homogenize it, followed by standing at room temperature for 12 hours. Subsequently, the suspension was centrifuged at 12,000 rpm for 15 minutes. Finally, the mixture was washed, resuspended and stored at 4°C.

### 2.4. Extraction of platelets

Platelets were isolated through a series of steps starting with the collection of whole blood from a male SD rats. First, anesthetize the rats, separate the skin and subcutaneous tissue along the sternum, open the thoracic cavity, expose the heart, press on the area where the heartbeat is most prominent, and use a blood collection needle to collect 10 ml of blood. The rats were euthanized immediately following blood sampling. Then, the whole blood samples were collected into anticoagulant tubes and left standing for 30 minutes. The test tube was centrifuged at 1000 rpm (cf1524r, Scilogex, USA) for 15 minutes, and the supernatant which contained PRP was collected. The supernatant was collected and centrifuged for 10 minutes at 3000 rpm. The supernatant was then discarded to obtain platelets which were mixed in 1 ml Phosphate-buffered saline (PBS, Cat. G4202, Servicebio, China) containing 2‰ concentration of prostaglandin (EI) to prevent platelet aggregation. Before use, it was centrifuged at 3000 rpm, and the sediment at the lower layer contained platelets. Take 200 μl of the lower suspension for later use, which contains a rich supply of platelets. [22].

### 2.5. Fabrication of HAMA hydrogel

The Hyaluronic Acid Methacryloyl (HAMA) was synthesized using a previously established protocol [23]. The Hyaluronic acid (HA, ZY9004, Shanghai Zeye Biological Technology Co., Ltd., China) was reacted to methacrylate leading to modification of the double bond. Specifically, HA was dissolved in water (1 g/100 ml). Subsequently, 1 ml of methyl methacrylate (MMA, 99%, Cat. M55909 Sigma-Aldrich, USA) was added to the solution, allowed to stand for 12 hours, and the potential of Hydrogen (pH) was adjusted within a range of 8–8.5. The entire process, including dissolution and reaction, was conducted on ice. At the end of the reaction, the solution was dialyzed for two days and then freeze-dried at – 40°C. Characterization of HAMA was performed with a Fourier-transform infrared spectrometer (FTIR, Nicolet is50, Thermo Fisher, USA). Fourier transform infrared spectrometry: freeze-dried HA/HAMA samples were mixed with Potassium Bromide and tabletted, which could be directly placed on Attenuated Total Reflection (ATR) accessories. The Fourier transform infrared spectrum of HA/HAMA was measured on the Infinity AR60 spectrometer. The spectrum recorded 64 scans between 450 and 4000 cm1 with a resolution of 2 cm-1. The HAMA was dissolved in a PBS solution with 3% w/v and 0.1% w/v of photoinitiator 2-Hydroxy-4’-(2-hydroxyethoxy)-2-methylpropiophenone (H137984, Aladdin, USA). The solution was exposed to ultraviolet light at 365 nm for 5 minutes, inducing the formation of hydrogel. Rheological measurements of the HAMA were conducted using a Rheometer (MARS 40, Thermo Fisher Scientific Inc, Waltham, MA, USA) equipped with parallel plates (diameter: 35 mm) to evaluate its rheological properties. The storage modulus (G’) and loss modulus (G“) of the HAMA were plotted on a logarithmic scale, where G’ < G” indicates a liquid state and G’ > G” indicates a solid state. The microstructures of HAMA hydrogels was characterized by the scanning electron microscope (SEM, model S-3400N, Hitachi, Tokyo, Japan).

### 2.6. Preparation of nanoparticle/plasmid complexes and bFGF/VEGFA@PRP hydrogel complexes

**Fig 1:**
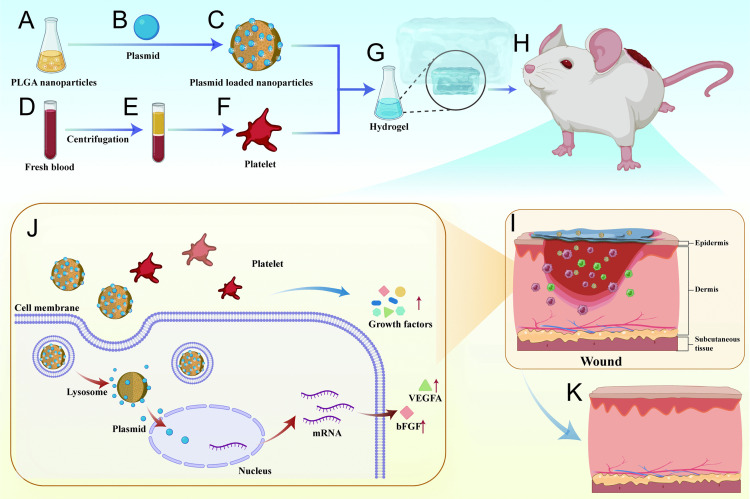
Schematic illustration of the preparation and application of bFGF/VEGFA@PRP hydrogel complexes for wound healing in rats. **(A)** PLGA nanoparticle. (B) bFGF and VEGFA plasmid. **(C)** Nanoparticle plasmid complexes. **(D)** Rat blood. **(E)** Blood samples were stratified after centrifugation. **(F)** Separate platelets. **(G)** Nanoparticles and platelets are mixed with hydrogel. **(H)** Wound model of full-thickness skin defect in rat back. **(I)** The bFGF/VEGFA@PRP hydrogel complex was applied to the wound of rats. **(J)** Complexes slowly release bFGF and VEGFA around the wound to promote angiogenesis of the skin and accelerate wound Healing in rats **(K)** Wound healed.

Nanoparticles and plasmids were assembled as depicted in Fig 1. Initially, 100 µg/µL polyethyleneimine (PEI) and a 10 mg/mL nanoparticle solution were added to distilled water. Subsequently, based on gel retardation assay results, the PEI-nanoparticle solution was mixed with plasmid at an N/P ratio of 6:1 (the ratio of the mole number of PEI amino group to the mole number of DNA phosphate group) and stirred uniformly [24]. The nanoparticle/plasmid complexes were formed through a process of electrostatic assembly, where the positively charged nanoparticles interact with the negatively charged plasmid DNA. This interaction leads to the encapsulation of the plasmid within the nanoparticle matrix. Nanoparticles/pEGFP complexes were incubated at 25 ◦C for 30 minutes. followed by the addition of the prepared nano-complex and PRP to the HAMA hydrogel to form the bFGF/VEGFA@PRP hydrogel.

### 2.7. Characterization of nanoparticle/plasmid complexes

The membrane vesicles of nanoparticle/plasmid complexes were examined by the SEM. Zetasizer Nano ZS (Malvern Instruments, Malvern, UK) was used to record the zeta potential of the resulting nanoparticles, PEI-nanoparticles, nanoparticle/plasmid complexes at a scattering angle of 90. The average hydrodynamic diameters of nanoparticles of nanoparticles, Nanoparticle/plasmid complexes were measured through dynamic light scattering (DLS, Brookhaven Instruments).

### 2.8. Release in vitro

To assess the capacity of bFGF/VEGFA@PRP hydrogel complexes for plasmid release, these complexes were first incubated in a PBS solution (pH 7.2) containing 0.02% w/v sodium azide. The incubation took place in a temperature-controlled incubator set at 37 °C. Buffer exchanges occurred on the 1st, 3rd, 5th, 7th, 14th, 21st, and 28th days. At each interval, the previously utilized PBS buffer was collected, and fresh PBS buffer was introduced. The samples were then stored at −80 °C, and the quantity of released plasmid was quantified using an enzyme-linked immunosorbent assay (ELISA) (BioOcean, Shoreview, MN).

### 2.9. In vivo and vitro transfection efficiency

Three rats were randomly selected to make a full-thickness skin defect model, and 1 ml of compound hydrogel was sucked by an empty needle syringe, which was applied externally to the wound made before and exposed for treatment. As mentioned above, the transfection efficiency of damaged skin wound is the highest one week after treatment with nanoparticle complex [25]. After one week, the three rats were euthanized, and the skin tissue around the wound and normal skin tissue were taken, fixed with paraformaldehyde, dehydrated with sucrose, and sliced into 10-µm sections. These sections were placed on slides, rinsed with PBS, stained with 4′,6-diamidino-2-phenylindole, and observed under the microscope. Take the skin tissue that was cut during wound creation and cut it into pieces. Spread the pieces evenly in a culture bottle and add a small amount of Dulbecco’s Modified Eagle Medium (DMEM, 11965092, Gibco, USA) containing 20% Fetal Bovine Serum (FBS, A3161001C, Gibco, USA). Let it stand at 37 °C for 4 hours. After the tissue attaches, supplement the culture medium and place the bottle in incubator for culture (37 °C, 5% CO2). On the third day, fibroblasts crawled out from the tissue edges. After the cells reached 80% confluence, they were passaged, and the remaining tissue blocks were filtered. The passaged cells were seeded into a six-well plate at a density of 1 x 10^5 cells per well. Subsequently, after the cells adhered to the wall, pEGFP-N 1 was added to each well to promote transient transfection. After 24 hours of culture, the cells were repeatedly washed with PBS and observed under a fluorescent microscope**.** The successfully transfected cells will have green fluorescence.

### 2.10. Grouping of experiments

Fifteen rats used for modeling were randomly assigned into 5 groups (n = 3). Immediately after establishment of the model, hydrogels containing different components (Table 1) were externally applied to the wound surface of rats with an empty needle syringe as follows and exposed for treatment. Each rat was kept in a separate cage and given adequate food and water.

**Table 1:** Grouping of experimental rats.

Groups	Complexes
NC group	1 ml of saline
NP group	1 ml of hydrogel
PRP group	1 ml of PRP- hydrogel (20 μl of PRP)
BV group	1 ml of bFGF/VEGFA hydrogel complex (5 μg of bFGF, 5 μg of VEGFA)
BVP group	1 ml of bFGF/VEGFA@PRP hydrogel complexes. (5 μg of bFGF, 5 μg of VEGFA and 10 μl of PRP)

### 2.11. Wound healing rate

The morphology of the wound tissue was recorded using the same camera at the same angle and distance at 0, 3, 9, and 15days post-injury under anesthesia. The wound area was measured by ImageJ software.

Percent wound closure (%) = [(Initial wound area – Wound area at time t)/ Initial wound area] × 100 [1].

### 2.12. Blood perfusion imaging

The blood flow in the wound was recorded on day 7 and day 15 using a Laser Doppler Flowmeter (MoorFLPI-2, USA). Rats were anesthetized with 50 mg/kg 2% pentobarbital sodium to allow measurement under anesthesia and avoid experimental errors caused by rat activity. The probe was placed 10 cm above the wound, and the signal intensity of blood flow in the wound was recorded. Subsequently, we used Laser Doppler Perfusion Imaging analysis software to analyze images.

### 2.13. H&E staining

After 15 days of treatment, the rats were euthanized, and the skin tissue around the wound was collected, fixed in 4% neutral-buffered paraformaldehyde for 24 h, dehydrated through a graded ethanol series (70%, 80%, 95%, 100%, 10 min each), cleared in xylene (2 × 15 min), and embedded in paraffin. Serial 5-µm sections were cut on a rotary microtome (Leica RM2235), floated on a 42 °C water bath, and mounted on positively charged slides. After drying at 60 °C for 1 h, sections were dewaxed in xylene (2 × 5 min) and rehydrated through descending ethanol (100%, 95%, 80%, 70%, 3 min each) to distilled water. Hematoxylin and eosin (H&E) staining was performed with the H&E staining kit (GB23303 Sevier Biotech, China) strictly following the manufacturer’s protocol: nuclei were stained with Mayer’s hematoxylin for 5 min, rinsed in running tap water for 3 min, differentiated in 1% acid ethanol for 3 s, and blued in 0.2% ammonia water for 30 s. After a brief rinse, cytoplasmic and extracellular components were counter-stained with eosin Y for 2 min. Slides were then dehydrated (70%, 80%, 95%, 100%, 2 min each), cleared in xylene (2 × 3 min), and mounted with neutral balsam. Then observed under an optical microscope.

### 2.14. Masson’s trichrome staining

Tissue sections (5 μm) were deparaffinized and rehydrated as described above. Masson’s trichrome staining was performed with the Masson Trichrome Staining Kit (G1346 Solarbio, China) following the manufacturer’s protocol: nuclei were stained with Weigert’s iron hematoxylin for 10 min, rinsed in running water for 2 min, and counter-stained with Acid Fuchsin solution for 5 min. Sections were then differentiated with phosphomolybdic-phosphotungstic acid solution for 5 min, transferred directly to Aniline Blue solution for 5 min, and rinsed briefly in 1% acetic acid for 30 s to remove excess dye. Slides were dehydrated quickly through 95% and 100% ethanol (5 s each), cleared in xylene (2 × 3 min), and mounted with neutral balsam. Collagen fibers stained blue, while cytoplasm and muscle appeared red to pink. Then observed under an optical microscope.

### 2.15. Immunohistochemistry

Immunohistochemistry (IHC) was employed to applied to measure the contents of bFGF and VEGFA. To further examine the collagen deposition in skin wound tissue, Collagen Type I (COL I) and Collagen Type III (COL III) were labeled and subjected to IHC. In addition, IHC labeling of platelet and endothelial cell adhesion molecule 1 (CD31), a marker of vascular endothelial cell, was conducted. To further monitor the proliferation and apoptosis of wound skin tissue cells, IHC labeling of marker of proliferation Ki67 and anti-apoptotic protein Bcl2 was performed. The paraffin slices were baked for 60 minutes, dewaxed and hydrated. The slide was put into citric acid buffer (PH 6.0) for 10 minutes, and then incubated with 3% hydrogen peroxide at 25°C for 10 minutes to block endogenous peroxidase. TBS (Tris-buffered saline: 50 mM Tris-HCl, 150 mM NaCl, pH 7.4) for all washing steps and antibody dilutions. Anti-bFGF antibody (bsm-34016R, Bioss, China, dilution 1:250), anti-VEGFA antibody (ab46154, Abcam, UK, dilution 1:250), anti-CD 31 antibody (28083–1-AP, Proteintech, USA, dilution 1:250), anti-Ki 67 antibody (ab209897, Abcam, UK, dilution 1:250), anti-Bcl 2 antibody (26593–1-AP, Proteintech, USA, dilution 1:250), anti-Collagen Type I antibody(HY-P81227, MCE, USA, dilution 1:250), anti- Collagen Type III antibodies (HY-P81101, MCE, USA, dilution 1:250) were added to the slices and incubated overnight. The slices were washed with TBS three times, followed by incubation with the second antibody at 37°C for 30 minutes. The color was developed using 3−3 ‘- diaminobenzidine (BNN2338, Sigma Aldrich, USA) for about 5 minutes, washed with TBS, and counterstained with hematoxylin for 30 seconds. After dehydration, the cover glass was sealed with neutral gel (N116470, Aladdin, USA) and dried. Finally, the slides were examined under a microscope. Semi-quantitative analysis was performed using ImageJ software.

### 2.16. Statistical analysis

Statistical analysis was performed using SPSS 22.0 software (IBM Company, Armonk, New York, USA). Quantitative analysis of experimental images was conducted using Image J software. Statistical significance was compared by One-way Analysis of Variance (ANOVA) and Least Significant Difference (LSD) comparison test. All experiments were performed in triplicate (n = 3). P < 0.05 was considered statistically significant. (Ns, no significant difference (P > 0.05); *, P < 0.05; **, P < 0.01; ***, P < 0.001).

## 3. Results

### 3.1. Characterization of PRP, nanoparticles, hydrogel and bFGF/VEGFA@PRP hydrogel complex

Platelets extracted from 10 ml of blood are resuspended in 1 ml of PBS. The platelet concentration measured by an automatic blood cell analyzer (Mindray, BC-10) is 2.402 x 10^12/L, with low concentrations of white blood cells (0.1 x 10^9/L) and red blood cells (1 x 10^10/L) (Table 2). Prior to use, PRP is centrifuged and resuspended, resulting in a platelet concentration of approximately 1.2 x 10^13/L.

**Table 2:** Cell composition in the extracted PRP.

Ingredient	Quantity	Unit
WBC (White blood cells)	0.10	*10^9/L
RBC (Red blood cells)	0.01	*10^12/L
PLT (Platelets)	2402.0	*10^9/L

**Fig 2:**
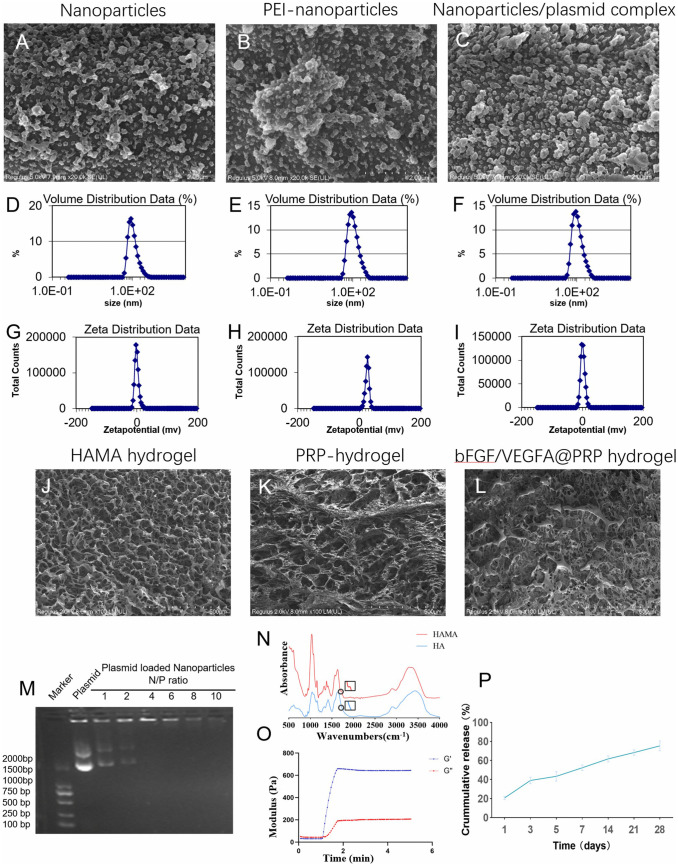
Characterization of Nanoparticles and Hydrogels. **(A)** SEM images of the nanoparticles. **(B)** Representative SEM images of the PEI-nanoparticles. **(C)** Representative SEM images of the nanoparticles/plasmid complexes. **(D)** The distribution of the hydrodynamic diameter of the nanoparticles (129.4 nm). **(E)** The distribution of the hydrodynamic diameter of the PEI-nanoparticles (136.2 nm). **(F)**The distribution of the hydrodynamic diameter of the nanoparticles/plasmid complexes (138.6 nm). **(G)** The Zeta potential of the nanoparticles (−0.817mV).(H) The Zeta potential of the PEI-nanoparticles (25.4mV). **(I)** The Zeta potential of the nanoparticles/plasmid complexes (1.09mV). **(J)** The representative SEM images of the HAMA hydrogel. **(K)** The representative SEM images of the PRP-hydrogel. **(L)** The representative SEM images of the bFGF/VEGFA@PRP hydrogel. **(M)** Agarose gel electrophoresis assay of the complexes at different N/P ratios. **(N)** In vitro release spectrum of the plasmid coated with nanoparticles in pH 7.2 PBS. **(O)** Characterization of HAMA was performed with a Fourier-transform infrared spectrometer. The stretching band of the C––C bond from methacrylate at 1700 cm − 1 was detected in HAMA. **(P)** Changes in the modulus of the HAMA after after being exposed to ultraviolet light at 365 nm for 5 minutes..

The nanoparticle/plasmid complexes was prepared according to the procedure illustrated in the flow chart(Fig 1A-G). Then the morphology of the complexes was examined by the SEM (Fig 2A-C). The composite appeared spherical and it was larger than that of nanoparticles and PEI-modified nanoparticles (Fig 2D-F), which allowed the effective packaging of nanoparticles/plasmid complexes. The average particle size of the composite was 138.6 nm by DLS (Fig 2F), which was lager than nanoparticles (Fig 2D, 129.4 nm) and PEI-nanoparticles (Fig 2E, 136.2 nm). The zeta potential of the nanoparticles PEI-nanoparticles and nanoparticle/plasmid complex were approximately −0.817mV, 25.4mV and 1.09 mV respectively (Fig 2G-I).

The pore structure of the bFGF/VEGFA@PRP hydrogel composite is presented in the SEM images (Fig 2J-L). The optimal nitrogen-to-phosphate (N/P) ratio was determined via gel electrophoresis mobility shift assays. At an N/P ratio of 6:1—calculated as the molar ratio of PEI amine groups to DNA phosphate groups—nanoparticles exhibited complete electrophoretic retardation of plasmid DNA, representing the maximal loading capacity (Fig 2M). The stretching band of the C––C bond from methacrylate at 1700 cm − 1 was detected in HAMA (Fig 2N).

In order to determine whether HAMA can solidify after being exposed to ultraviolet light at 365 nm for 5 minutes, we measured the modulus of HAMA after treatment. The results show that HAMA changes from liquid to solid after being exposed to ultraviolet light (Fig 2O).

### 3.2. In vitro release of nanoparticles

The in vitro release profile of the nanoparticles was evaluated (Fig 2P). On the third day, over 40% of the plasmid was released from the nanoparticles, while the release rate exceeded 50% by the seventh day. Furthermore, approximately 75% of the plasmid was released gradually and steadily until the 28th day. These findings provide evidence of the sustained release capability of the nanoparticles.

### 3.3. Transfection efficiency in vivo

**Fig 3:**
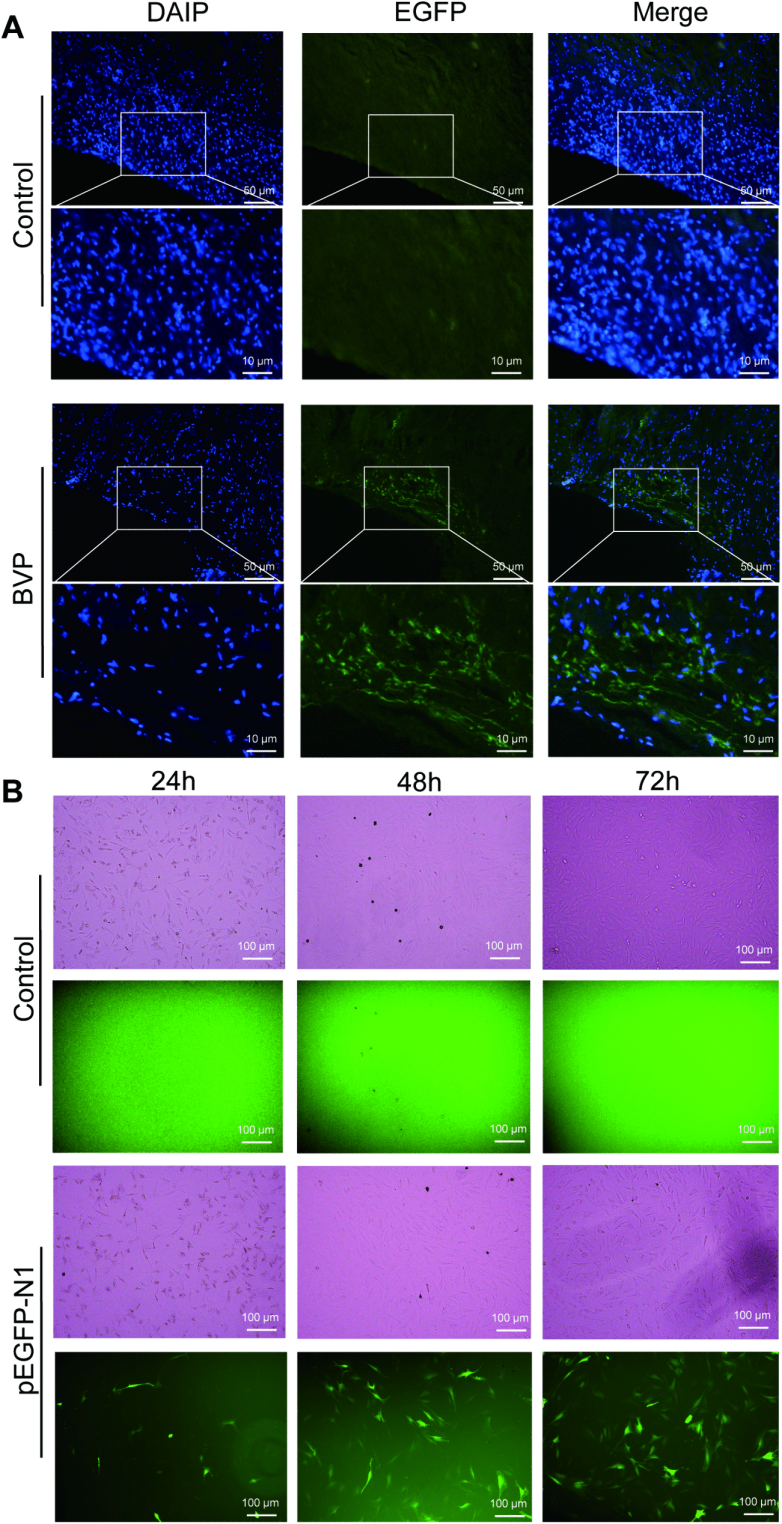
Expression of nanoparticles transfected with pEGFP-N1 in rats in vivo and in vitro. **(A)** Distribution and transfection efficiency of bFGF/VEGFA-loaded nanoparticles in rat skin tissue. Representative fluorescence microscopy images of skin sections from control and BVP (bFGF- and VEGFA-loaded nanoparticles) groups. In the control group, no detectable fluorescence signal was observed, indicating the absence of nonspecific background fluorescence. In contrast, strong fluorescence expression was detected in the skin tissue of the BVP-treated group, demonstrating successful transfection and localized delivery of the nanoparticles carrying bfgf and VEGFA genes. **(B)** Time-course analysis of EGFP expression in cells following pEGFP-N1 transfection. Cells were transfected with the pEGFP-N1 plasmid and monitored at 24, 48, and 72 h post-transfection. Green fluorescence intensity and the number of EGFP-positive cells increased progressively from 24 to 72 h post-transfection, paralleling cell proliferation and expansion. These observations suggest that the enhanced EGFP signal resulted from both sustained transgene expression and the increasing cell population, demonstrating successful transfection and stable maintenance of the exogenous reporter gene during cell culture expansion.

Skin tissue samples were collected one week post-application of the bFGF/VEGFA@PRP hydrogel. EGFP expression was assessed to determine transfection efficiency. Results demonstrated significant fluorescence in damaged skin tissue treated with the compound (Fig 3A), indicating successful hydrogel transfection in rats.

### 3.4. Transfection efficiency in vitro

The expression of green fluorescent protein in cells treated with pEGFP-N 1 was detected (Fig 3B). The green fluorescence in the pEGFP-N 1 treatment group indicate positive expression, confirming successful transfection. Green fluorescence intensity and the number of EGFP-positive cells increased progressively from 24 to 72 h post-transfection, paralleling cell proliferation and expansion.

### 3.5. Wound healing

**Fig 4:**
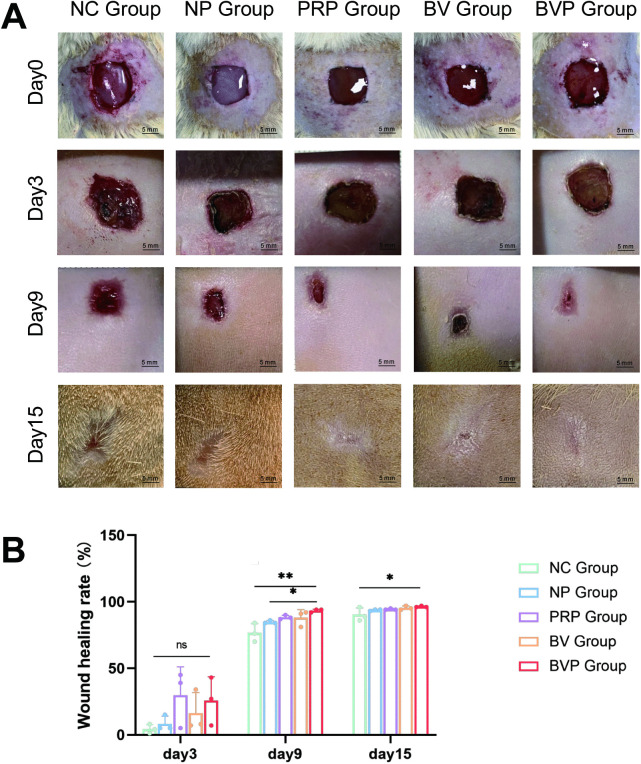
Accelerated wound closure by bFGF/VEGFA@PRP hydrogel complexes in a full-thickness wound model. **(A)** Representative photographs of wound closure at 0, 3, 9 and 15 days post-wounding. **(B)** Quantification of wound closure expressed as percentage of initial wound area. Data are mean ± SD from three independent experiments (n = 3 rat per group). “NC” indicates wounds treated with 1 ml of saline only; “NP” indicates wounds treated with 1 ml of hydrogel. “PRP” indicates wounds treated with 1 ml of PRP-hydrogel (20 μl of PRP); “BV” indicates wounds treated with 1 ml of bFGF/VEGFA hydrogel complex (5 μg of bFGF, 5 μg of VEGFA); “BVP” indicates wounds treated with 1 ml of bFGF/VEGFA@PRP hydrogel complexes (5 μg of bFGF, 5 μg of VEGFA and 10 μl of PRP). Statistical significance was evaluated by one-way ANOVA and LSD comparison test. Ns, no significant difference (P > 0.05); *P < 0.05; **P < 0.01. Control at the same time point.

To investigate the morphology of skin wounds during the healing process in rats, images were recorded on days 0, 3, 9, and 15 post-injury (Fig 4A). On day 3 post-injury, the wounds started to heal by forming crusts and there was no significant exudation, except in the NC group. On day 9, the area of the wound was significantly reduced in all groups. The BVP group had the smallest trauma area, with some scabs falling off and the trauma becoming smooth. New epidermis and dermis tissues were formed in the BVP group, while partial defects were observed in the injured skin in other groups. On day 15, the wounds of all groups had satisfactorily healed. The statistical results of the wound healing rates among the groups are shown in Fig 4B. On day 9 after injury, the healing rate of the NC group was significantly lower than that of the other groups. The wound healing rate of BVP group was the highest (93%), compared to that of the NC group and the NP groups. (*P < 0.05; **P < 0.01). But there was no significant difference in wound healing rate between the BVP group and either the PRP or BV group. On day 15, the wound healing rate in the BVP treatment group was significantly higher than that in the control group (*P < 0.05).

### 3.6. Analysis of wound blood perfusion

**Fig 5:**
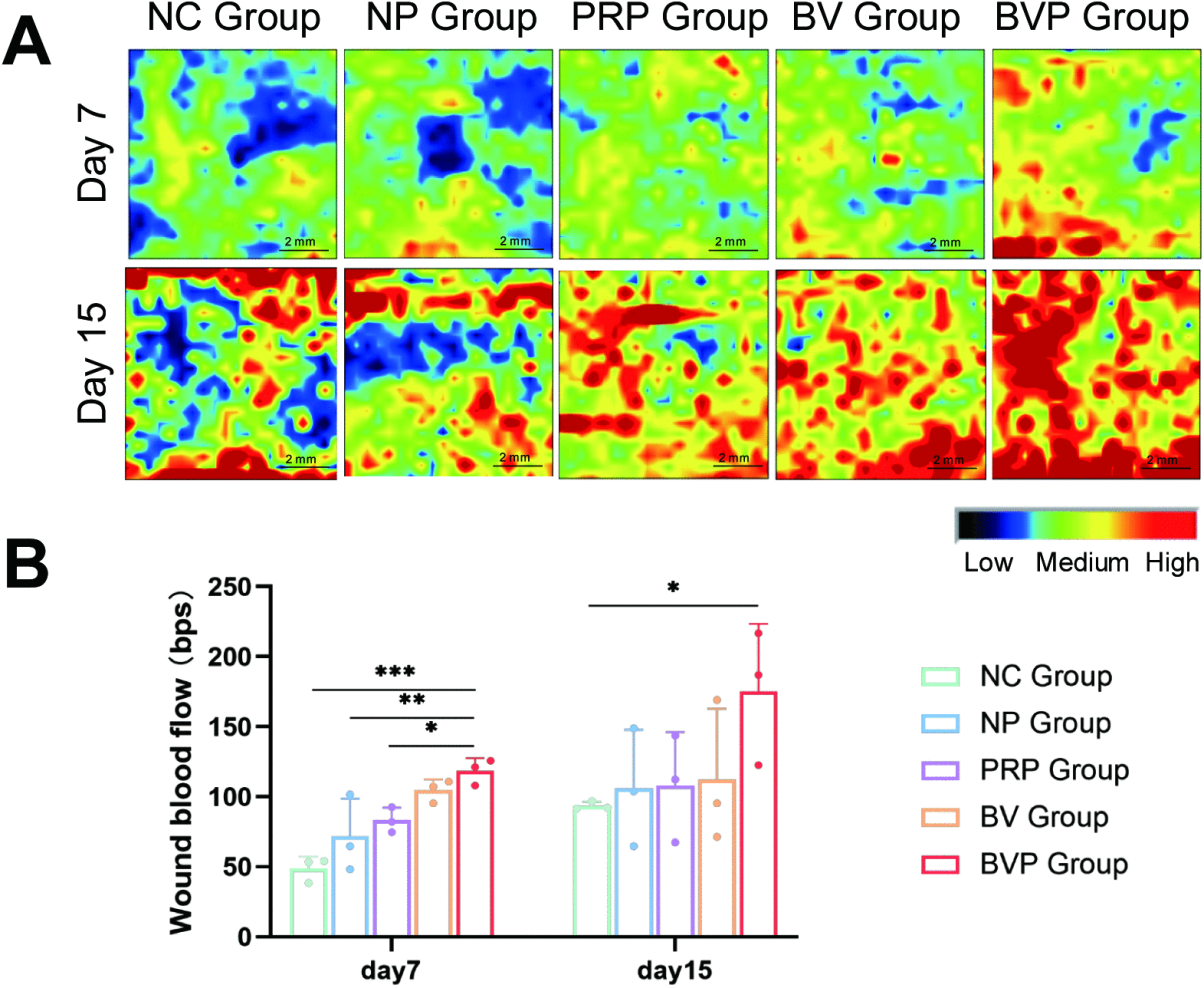
Enhanced blood perfusion in the wound area at 7 and 15 days post-wounding. **(A)** Representative laser-speckle images of wound perfusion at 7 and 15 days post-wounding; red denotes high and blue denotes low blood flow. **(B)** Quantification of relative blood perfusion (perfusion units, PU) in the wound area. Data are mean ± SD of three independent animals (n = 3 mice per group). “NC” indicates wounds treated with 1 ml of saline only. Statistical significance was evaluated by one-way ANOVA and LSD comparison test. *P < 0.05, **P < 0.01, ***P < 0.001. Control at the same time point.

The formation of new blood vessels contributes to tissue repair, especially following skin trauma [26]. On day 7 (Fig 5), the blood flow of wounds in each group was inconsistent, and the blood flow in BVP group was significantly higher than that in other groups (*P < 0.05; **P < 0.01; ***P < 0.001). On day 15, the blood flow signals increased signal in each group were enhanced, but the blood flow signal intensity in the BVP group was significantly higher than that in the NC group (*P < 0.05), while it in the PRP group and the BV group were slightly lower than in the BVP group.

### 3.7. H&E staining analysis

**Fig 6:**
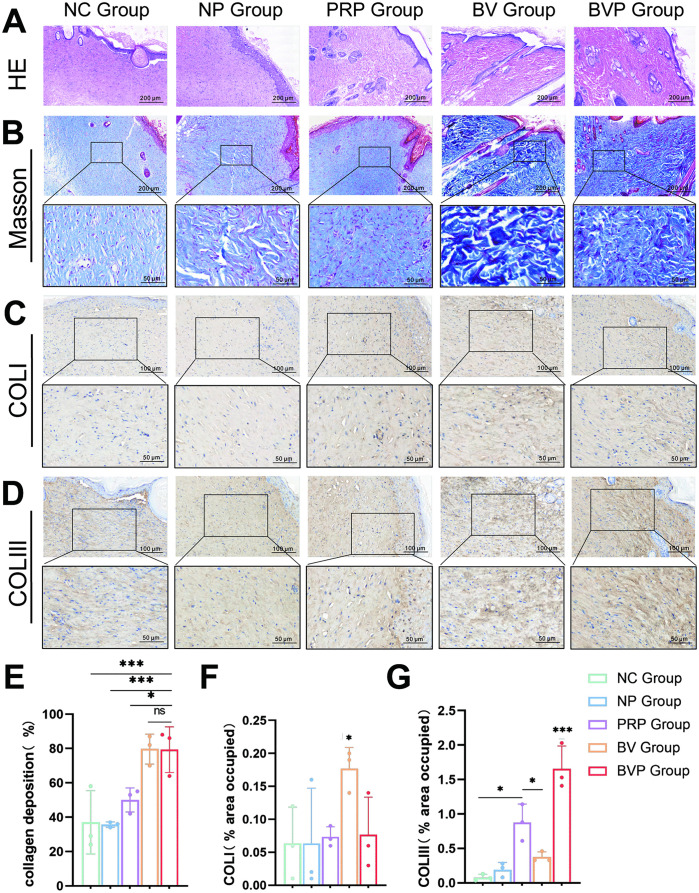
Histological evaluation and collagen deposition in wound tissue on day 15 post-wounding. **(A)** Representative Hematoxylin & eosin (H&E) staining images of wound sections; The red arrow refers to the skin appendage. **(B)** Representative Masson’s trichrome staining images of wound sections; blue color indicates collagen. **(C)** Representative immunohistochemical images of wound sections showing type-I collagen (COLI) expression. **(D)** Representative immunohistochemical images of wound sections showing type-III collagen (COLIII) expression. **(E)** Quantification of total collagen deposition (Masson-stained area %). **(F)** Quantification of COLI deposition (% positively stained area). **(G)** Quantification of COLIII deposition (% positively stained area). In E–G, data are mean ± SD from three independent animals (n = 3 mice per group). Statistical significance was evaluated by one-way ANOVA and LSD comparison test. Ns, no significant difference (P > 0.05); *P < 0.05, **P < 0.01, ***P < 0.001.

To gain a detailed understanding of the wound healing process, histological changes in the skin were examined (Fig 6A). H&E staining revealed a gradual detachment of scabs, complete wound closure, restoration of normal epidermal structure, and the formation of disorganized collagen fibers. On day 15, a significant accumulation of inflammatory cells under the epidermis was observed in the NC group, and there was uneven thickening of the regenerated epidermis [27,28]. Epidermal thickening was observed in the NP group, while the PRP group, BV group and BVP group showed enhanced production of some sebaceous gland. The BVP group had the most new sebaceous glands, hair follicles and blood vessels compared with the other groups.

### 3.8. Masson staining analysis

Collagen promotes cell proliferation and differentiation. However, excessive production of disordered collagen in the dermis promotes the formation of scars [29]. The MT staining was performed to examine the degree of collagen fibrosis (Fig 6 B). After 15 days of treatment, the collagen fibers in the skin of the NC group and NP group were dense and disordered. The collagen deposition in the BV group was the most significant, and the collagen fibers were arranged in large chunks, but the collagen fibers in the PRP group were significantly thinner than those in the BV group. Conversely, collagen deposition and distribution were significantly improved in the BVP group (Fig 6 E). On day 15, collagen density in the BV and BVP group was higher than that in other groups (**P < 0.01).

### 3.9. Immunohistochemical staining analysis

The deposition of collagen in the skin was examined by Immunohistochemistry. Results indicated that the bFGF/VEGFA group had the highest total content and type I collagen deposition (*P < 0.05) (Fig 6 C). The content of type III collagen in the BVP group was highest and there was significant difference with PRP group (***P < 0.001). The expression of COL III in the PRP group was higher than that in the NC group, NP group and BV group (*P < 0.05).

BCL 2 inhibits cell apoptosis, while Ki 67 is a cell-associated antigen that is involved in the regulation cell proliferation to accelerate wound healing. The expression of BCL 2 in the BV group was higher than that in the NC group, NP group and PRP group, but lower than that in the BVP group(*P < 0.05; **P < 0.01; ***P < 0.001). The expression of Ki 67 in the BVP group was also statistically different from other groups (**P < 0.01; ***P < 0.001).

**Fig 7:**
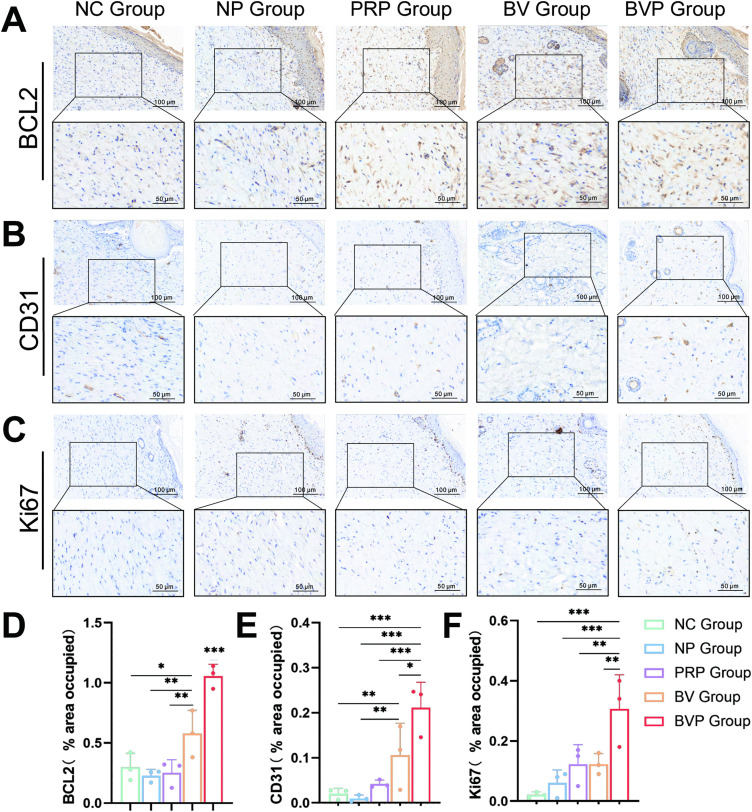
BCL2, CD31 and Ki67 expression in wound tissue on day 15 post-wounding. **(A)** Representative immunohistochemical images of wound sections showing B-cell lymphoma 2 (BCL2) expression; brown DAB signal indicates positive staining. **(B)** Representative immunohistochemical images of wound sections showing Endothelial cell marker CD31 expression. **(C)** Representative immunohistochemical images of wound sections showing Proliferation marker Ki67 expression. **(D)** Quantification of BCL2 positive staining area (% of total wound bed). **(E)** Quantification of CD31 positive staining area (% of total wound bed). **(F)** Quantification of Ki67 positive staining area (% of total wound bed). Data are mean ± SD from three independent animals (n = 3 mice per group). Statistical significance was evaluated by one-way ANOVA and LSD comparison test. *P < 0.05, **P < 0.01, ***P < 0.001.

Angiogenesis is important in wound healing, which provides sufficient nutrition and oxygen for the wound to promote granulation tissue formation [30]. To measure the immune response of angiogenesis, we performed IHC for the CD31 protein on the wound tissue 15 days after injury and carried out quantitative analysis (Fig 7 E). The CD 31 content in the BV group was higher than that in the NC group, NP group and PRP group (**P < 0.01), but there was no significant difference compared with the PRP group. Compared with the other groups, the expression of CD31 in the group treated with bFGF/VEGFA@PRP hydrogel was highest (*P < 0.05; ***P < 0.001).

**Fig 8:**
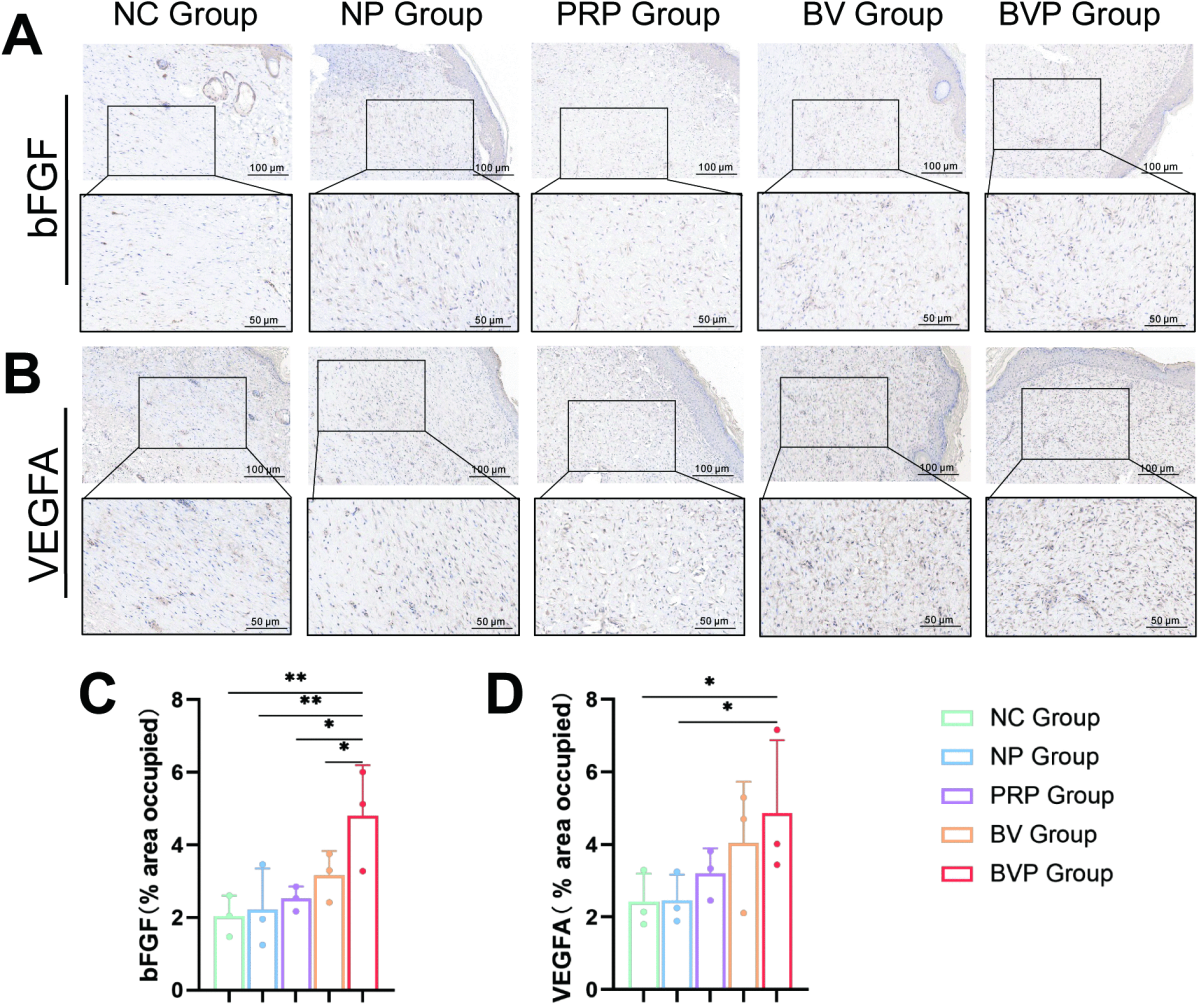
bFGF and VEGFA expression in wound tissue on day 15 post-wounding. (A) Representative immunohistochemical images of wound sections showing basic fibroblast growth factor (bFGF) expression. Brown DAB signal indicates positive bFGF staining. (B) Representative immunohistochemical images of wound sections showing Vascular endothelial growth factor A (VEGFA) expression. (C) Quantification of bFGF positive staining area (% of total wound bed). (D) Quantification of VEGFA positive staining area (% of total wound bed). Data are mean ± SD from three independent animals (n = 3 mice per group). Statistical significance was evaluated by one-way ANOVA and LSD comparison test. *P < 0.05, **P < 0.01, ***P < 0.001.

Semi-quantitative analysis of bFGF and VEGFA in skin tissues was performed using IHC (Fig 8). VEGFA levels in the BVP group differed significantly from those in the NP and NC groups (*P < 0.05), and it also better than the PRP group and BV group. Additionally, the BVP group exhibited significantly higher bFGF levels compared to the PRP group, BV group and other groups (*P < 0.05; **P < 0.01).

## 4. Discussion

Full-thickness skin defects are injuries that affect the epidermis, dermis, and their associated structures. The repair process involves complex mechanisms, including cell migration, angiogenesis, and matrix remodeling. Because these wounds lack residual skin appendages, such as hair follicles and sweat glands, they cannot regenerate through epithelialization alone. Furthermore, they often lead to pathological scar formation [31–34] and functional impairment, which makes clinical wound repair challenging. In recent years, advancements in tissue engineering and regenerative medicine have allowed researchers to overcome traditional treatment bottlenecks. By integrating biomaterials, stem cell technology, and genetic engineering, they have promoted innovative strategies for repairing full-thickness skin defects. However, the instability of materials, the tumorigenic risks associated with stem cell therapy, and the ethical concerns surrounding genetic engineering have hindered the clinical translation of current therapies.

In recent years, nanoparticles containing drugs have been developed to target and treat diseased tissues. This is considered as a major breakthrough in medical technology because it provides more robust and effective treatment. Therefore, nanoparticle materials are increasingly being used in wound healing [35]. Compared with other carriers, nanospheres have several advantages, including easy modification, better biocompatibility and rapid biodegradation [36]. Therefore, better strategies should be developed to accelerate the clinical application of nano-drug conjugates [37]. Some members of the bFGF and VEGFA family were reported to promote wound healing [38,39]. For instance, bFGF promoted angiogenesis in vivo and in vitro [40], cell growth and differentiation, granulation tissue and wound remodeling of new epithelial tissue [41]. The most important factor affecting the speed and quality of wound healing is angiogenesis [42]. Evidence from previous studies indicates that VEGFA promotes vascular repair of wounds in diabetic animals [43]. Growth factors such as bFGF and VEGFA can enhance wound closure, however, their short half-life (approximately 2–6 hours) and vulnerability to protease degradation require frequent dosing, which can lead to inconsistent effectiveness [44]. Recently, PRP has gained attention for its high levels of growth factors that come from the same individual (like bFGF and VEGFA) [45–47]. However, its rapid release—in which 80% of its active components are released within 72 hours—makes it hard to provide ongoing biological support during the later stages of healing [48]. Many studies have proven that PRP can act as a stimulator, effectively promoting the proliferation of fibroblasts and the secretion of growth factors such as bFGF and VEGFA [49,50]. Therefore, the bFGF and VEGFA were encapsulated by nanoparticles and combined with PRP and hydrogel to explore whether the bFGF/VEGFA@PRP hydrogel could play a role in the treatment of wounds. Compared to growth factor therapy and PRP therapy, can this new type of biological dressing achieve better results in the healing of full-thickness skin defects?

We first constructed nanoparticles/pEGFP containing bFGF and VEGFA plasmids (Fig 1A-C), and then mixed them with PRP into the hydrogel (Fig 1G), which was used for rat wounds (Fig 1H). The complexes adhered to the wound surface and slowly released bFGF and VEGFA to promote skin angiogenesis and accelerate wound healing (Fig 1I-K).

Characterization and analysis were conducted on the nanospheres and drug-loaded hydrogels. The positively charged surface of the nanospheres (Zeta potential +1.09 mV) (Fig 2A-I) protects plasmids from nuclease degradation through electrostatic adsorption. Additionally, it triggers gene-controlled release [51]. The loose and porous structure of the hydrogel enhanceed the adhesion of nanospheres to host cells (Fig 2J-L), further optimizing the transfection microenvironment. Gel retardation analysis revealed that nanoparticles achieved optimal DNA binding at an N/P ratio of 6:1 (PEI amine/DNA phosphate), as evidenced by complete migration inhibition of the plasmid (Fig 2M). And it provided physical support for cell adhesion, migration, and proliferation [52,53]. The presence of the characteristic C–C stretching vibration at 1700 cm ⁻ ¹ in HAMA, attributed to methacrylate groups, provided spectroscopic evidence for successful HA modification. This spectral signature indicated that methacrylate groups had been successfully conjugated to the HA chains [23]. Upon exposure to 365 nm UV light for a duration of 5 minutes, the methacrylate groups of HAMA underwent radical polymerization. Rheological characterization revealed a substantial increase in elastic modulus (≈644 Pa), confirming the transformation from a viscous liquid to an elastic solid state (Fig 2O). This super-soft hydrogel provides a moist environment for the wound, with almost no mechanical stimulation, and avoids the oppression of new granulation tissue [54].

Through our experiments, it can be observed that growth factors are rapidly released in the first 72 hours due to the expected rapid PRP burst [55], while in the subsequent 21 days, growth factors are released slowly, benefiting from the controlled release effect of the nanospheres [56]. Immunofluorescence shows that there are more EGFP-positive cells at the injury site, indicating that the complex has been successfully transfected in rats (Fig 3A). In vitro experiments also demonstrate that pEGFP-N1 has been successfully transfected and stably maintained in cells (Fig 3B).

On day 15, the wounds of rats in all groups are essentially healed (Fig 4A), and there was a significant difference between the BVP group and NC group. This shows that the bFGF/VEGFA@PRP hygrogel complex has a positive effect on wound healing. Mechanistically, the active growth factors and bioactive substances in PRP are released rapidly in the early stage of treatment (acute injury stage), playing a role in rapid hemostasis, anti-inflammation and reducing early tissue injury; the plasmid DNA encoding bFGF and VEGFA is released slowly and sustainably in the middle and late stages of treatment (cell proliferation and tissue regeneration stage), realizing long-term stable local gene transfection, continuously inducing the expression of target growth factors, and promoting cell proliferation, migration and angiogenesis. There was no significant difference in wound healing rate between the BVP group and either the PRP or BV group.. We speculate that the gene expression process takes time before its effects on the wound surface can be observed [25]. Limitations include the relatively small sample sizes and the small wounds, both of which confounded our results. The results of blood perfusion measurements showed that the BVP group showed improved blood flow in the wound skin on the 7th and 15th days of healing compared to the other groups, demonstrating that bFGF/VEGFA@PRP hydrogel effectively repaired the damaged blood vessels and promoted the reconstruction of blood flow in damaged skin (Fig 5). Results of the HE staining experiments showed that the BVP treatment group and the PRP group had significantly increased appendages in the healed skin(Fig 6A).

To clarify the effect of the compound on wound remodeling, we conducted Masson staining. After 15 days of treatment, Masson staining showed that collagen deposition was higher in the BVP groups, which shows that the compound promotes wound healing by promoting collagen deposition(Fig 6B, E). However, in the BV group, collagen was arranged in dense chunks and unevenly distributed, whereas in the BVP group, collagen deposition and distribution were more uniform. The blue staining intensity was lower in the BVP group compared to the BV group, with collagen fibers appearing thinner and more loosely arranged. It may be because wound healing is promoted with reduced scarring by the release of PRP [57].

To further investigate collagen deposition, we measured and quantified collagen type I and type III in the skin using immunohistochemistry(Fig 6C, D). Collagen’s cross-linking characteristics play a crucial role in determining scar-free wound healing: excessive deposition of type I collagen during wound healing leads to scar formation. Type III collagen is smaller and contributes to the softness and elasticity of the skin [58]. It was observed that bFGF/VEGFA nanoparticles promoted wound healing, cell proliferation, type I collagen deposition, while when applied in combination with PRP, it downregulated type I and upregulated type III collagen deposition, accelerated wound healing and enhanced the elasticity of the skin. This study demonstrates that the addition of PRP increased type III collagen and decreased type I collagen, suggesting that further research is essential to understand its implications.

To elucidate the therapeutic effects of the bFGF/VEGFA@PRP hydrogel complex, we performed research at the molecular level. IHC examination revealed that the expression level of CD 31 molecules was higher in the BVP group than in PRP group(Fig 7B, 7E), BV group and other groups, which was due to the release of growth factors by bFGF/VEGFA plasmid and PRP co-expresses with it. There were significant difference between PRP group and BV group and the control group, probably because bFGF and VEGFA promoted angiogenesis, providing sufficient oxygen and nutrients supply to sustain tissue regeneration and facilitate the healing of damage tissue [59]. The BVP group showed a significant increase in the expression of BCL 2 and Ki 67 compared to the other groups(Fig 7A, C, D, E). The above results indicate that the bFGF/VEGFA@PRP hydrogel complex may accelerate the wound healing process by inhibiting apoptosis, and enhancing cell proliferation. Notably, a significant increase in bFGF and VEGFA expression in the BVP group (Fig 8). We speculate that the therapeutic effect of the drug is achieved through two aspects: 1. The direct release of growth factors by PRP; 2. The sustained release effect of bFGF/VEGFA nanoparticles on growth factors.

In conclusion, our animal models demonstrated that the bFGF/VEGFA@PRP hydrogel complex effectively promoted wound healing by releasing growth factors, enhancing angiogenesis, increasing collagen deposition, boosting cell proliferation, and inhibiting apoptosis.

This article verifies the advantages of the bFGF/VEGFA@PRP hydrogel complex in promoting wound healing and collagen deposition. Furthermore, the effect of PRP combined with bFGF/VEGFA nanospheres is significantly better than that of a single treatment. The growth factors released early from the PRP quickly initiate the repair process. Meanwhile, the sustained release of gene plasmids from the nanospheres allows fibroblasts and endothelial cells at the wound edge to continuously express bFGF/VEGFA proteins. This overcomes the limitation of traditional PRP treatment, where growth factor activity lasts only 72 hours [60]. This dual-phase approach of “initial stage activation and later stage maintenance” aligns well with the changing demands for growth factors during skin repair. Nevertheless, superior healing outcomes observed in the BVP group are regarded as merely additive compared with the effects of PRP or BV nanoparticles alone. The benefit of the combination arises from the cumulative, rather than supra-additive, contributions of PRP-mediated pro-angiogenic signaling and BV nanoparticle-mediated gene delivery.

However, certain limitations remain. The present work does not include in-vitro release profiles of individual PRP-derived factors (e.g., VEGFA, bFGF). While this study establishes the formation and mechanical integrity of HAMA hydrogels, their long-term degradation kinetics under physiological conditions remain to be fully characterized. Consequently, although we repeatedly refer to the ‘early burst’ of PRP as a limitation, this remains a conceptual, rather than experimentally quantified, drawback. These include the need to increase the sample size in real situations and the necessity to optimize the observation period. Although no significant immune rejection reactions were observed in the combination treatment group, the long-term biosafety of the gene nanoballs, including the risk of plasmid integration, needs validation through subsequent studies such as whole-genome sequencing.

## 5. Conclusion

We successfully prepared a hydrogel composite containing gene nanospheres and PRP, overcoming the limitations of traditional platelet-rich plasma (PRP) treatment. This study shows that the bFGF/VEGFA@PRP hydrogel complex effectively promotes wound healing by releasing growth factors, enhancing angiogenesis, increasing collagen deposition, boosting cell proliferation, and inhibiting apoptosis. Nevertheless, it presents shortcomings, including a shortage of platelets and unresolved issues regarding the long-term biosafety of gene nanoballs that require further investigation.

## Supporting information

S1 Fig Raw_images. (TIF)
