# Supplementary figures and images for: A bioactive hydrogel integrating bFGF/VEGFA gene-loaded nanoparticles and platelet-rich plasma for accelerated full-thickness skin wound healing
Source: PLoS One. 2026 May 26;21(5):e0350087. doi: 10.1371/journal.pone.0350087 (PMC13210390; doi:10.1371/journal.pone.0350087)

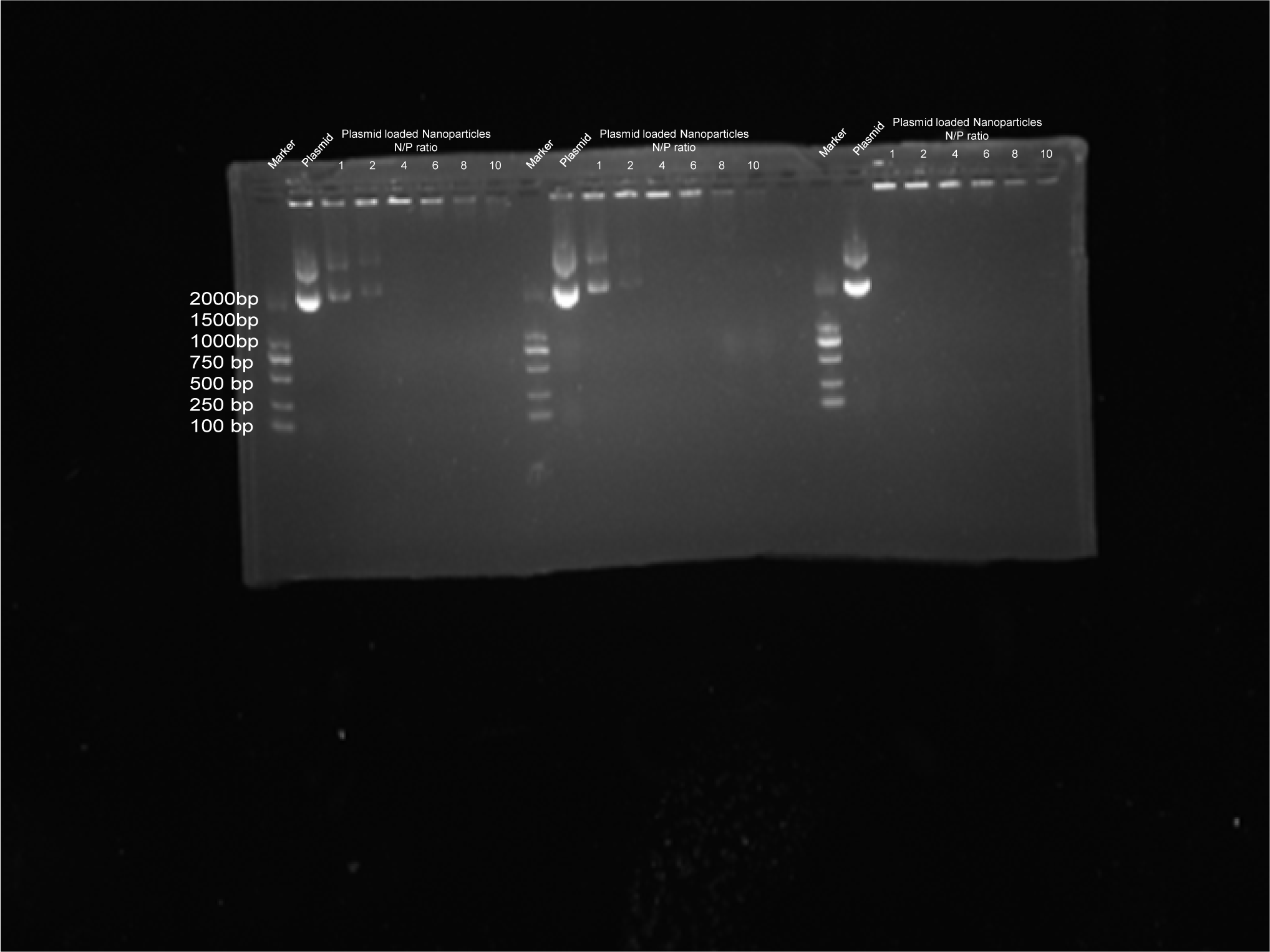

Supplement: S1 Fig — (TIF) [file pone.0350087.s001.tif]
